# The Novel Nrf2 Activator Omaveloxolone Regulates Microglia Phenotype and Ameliorates Secondary Brain Injury after Intracerebral Hemorrhage in Mice

**DOI:** 10.1155/2022/4564471

**Published:** 2022-03-11

**Authors:** Libin Hu, Yang Cao, Huaijun Chen, Lei Xu, Qiguo Yang, Hang Zhou, Jianru Li, Qian Yu, Zhangqi Dou, Yin Li, Feng Yan, Fuyi Liu, Gao Chen

**Affiliations:** ^1^Department of Neurosurgery, The Second Affiliated Hospital of Zhejiang University School of Medicine, China; ^2^Zhejiang University School of Medicine, China

## Abstract

The polarization of microglia is recognized as a crucial factor in reducing neuroinflammation and promoting hematoma clearance after intracerebral hemorrhage (ICH). Previous studies have revealed that redox components participate in the regulation of microglial polarization. Recently, the novel Nrf2 activator omaveloxolone (Omav) has been validated to improve neurological function in patients with neurodegenerative disorders by regulating antioxidant responses. In this study, we examined the efficacy of Omav in ICH. Omav significantly promoted Nrf2 nuclear accumulation and the expression of HO-1 and NQO1 in BV2 cells. In addition, both *in vitro* and *in vivo* experiments showed that Omav treatment inhibited M1-like activation and promoted the activation of the M2-like microglial phenotype. Omav inhibited OxyHb-induced ROS generation and preserved the function of mitochondria in BV2 cells. Intraperitoneal administration of Omav improved sensorimotor function in the ICH mouse model. Importantly, these effects were blocked by pretreatment with ML385, a selective inhibitor of Nrf2. Collectively, Omav modulated microglial polarization by activating Nrf2 and inhibiting ROS generation in ICH models, suggesting that it might be a promising drug candidate for the treatment of ICH.

## 1. Introduction

Intracerebral hemorrhage (ICH), which is mainly caused by rupture of blood vessels in the brain, inflicts 10-30 per 100000 population each year [1]. In addition to surgical removal of the hematoma, limited medical treatments are effective at improving neurological functional recovery [2, 3]. Brain injury caused by ICH includes primary injury and secondary injury. Primary brain injury is mainly caused by direct mechanical effects of the hematoma, while secondary brain injury is mediated by blood components, activation of microglia/macrophages, reactive oxygen species (ROS) generation, and cytokine release. Based on accumulating evidence, secondary brain injury strongly contributes to neurological deficiency [4]. However, to date, few treatments for secondary injury have proven beneficial for improving the prognosis.

Microglia, as brain-resident macrophages of the immune system, are activated by exudative blood components to further participate in inflammation and hematoma clearance. Microglia are endowed with spectacular plasticity, which allows them to acquire different phenotypes and exert multiple effects on tissue damage and repair [5]. Typically, the activation state of microglia exhibits a spectrum of proinflammatory (M1) or alternative (M2) responses [6, 7]. M1 microglia secrete proinflammatory cytokines (IL-1*β* and TNF-*α*) and induce the production of ROS, which subsequently amplify M1 polarization and result in neuronal injury [8]. M2 microglia, which are mainly polarized in the subacute and chronic phase, are important for resolving the hematoma and wound healing and are generally characterized by the expression of factors such as CD206, arginase (Arg-1), and chitinase-like protein 3 (known as Ym1) [6, 7, 9, 10]. Published evidence indicates that dysregulated microglial activation leads to a deleterious and neurotoxic microglial phenotype, which is defined by prolonged inflammation and inefficient hematoma clearance [11–13]. The balance of microglia polarization is currently postulated to be beneficial for functional recovery after ICH [14, 15].

Accumulating evidence indicates a crucial role for redox components in microglial polarization [16, 17]. Nuclear factor erythroid-2 related factor 2 (Nrf2) is a key transcription factor and regulator of antioxidant responses that exerts a protective effect after ICH [17–19]. Once activated, Nrf2 translocates to the nucleus and binds to the antioxidant response element (ARE) to initiate the expression of antioxidant enzymes and proteins such as heme oxygenase-1 (HO-1), NAD(P)H: quinone oxidoreductase-1 (NQO1), and superoxide dismutase (SOD). Recent studies have reported that Nrf2 knockout (Nrf2-/-) mice presented higher ROS levels, leukocyte infiltration, and larger injury volumes that correlated with more severe neurological deficits in the post-ICH period [19]. Moreover, the upregulation of HO-1 contributes to a reduction in ROS accumulation, which was the main inducer of secondary injury after ICH [8]. Selective suppression of mitochondrial ROS production inhibits microglial M1 polarization and attenuates secondary brain injury [11]. In recent years, the Nrf2 pathway has attracted increasing attention as a potential target for oxidative stress-related CNS disease. Monascin, an Nrf2 activator, has been shown to facilitate hematoma clearance and attenuate iron overload after experimental ICH [20]. Another Nrf2 activator, RS9, exerts its neuroprotective effect on ICH by upregulating HO-1 and SOD-1 expressions [21]. Nevertheless, the safety, pharmacokinetics, and pharmacodynamics of monascin and RS9 remain unclear in both mouse models and humans.

Omaveloxolone (Omav) is a novel synthetic oleanane triterpenoid compound that is currently being evaluated in clinical trials for the therapy of Friedreich ataxia (FA). Clinical trials have shown that Omav significantly improves the neurological function of patients with FA [22, 23]. In addition, Omav exerts a pharmacological effect on the treatment of mitochondrial diseases and seizures [24, 25]. Based on these studies, Omav exerts antioxidant and anti-inflammatory effects by activating Nrf2 and inducing the transcription of cytoprotective genes. In conclusion, Omav has the potential to play an important role in the treatment of redox-related CNS diseases. Hence, this study focused on determining the effect of Omav on microglial polarization after ICH and the relationship between these effects and neurological functional recovery.

## 2. Materials and Methods

### 2.1. Ethics Statement

The animal study was reviewed and approved by the Institutional Ethics Committee of the Second Affiliated Hospital, Zhejiang University School of Medicine.

### 2.2. Animals and Grouping

All mice were obtained from SLAC Laboratory Animal Co., Ltd. (Shanghai, China) and housed in a temperature- and humidity-controlled environment on a 12-hour light/dark cycle with free access to water and food. The present study was conducted according to the National Institutes of Health Guide for the Care and Use of Laboratory Animals.

Mice were randomly assigned to the sham group, ICH+vehicle group, ICH+Omav group, and ICH+Omav+ML385 group.

### 2.3. ICH Model

The autologous blood injection model of ICH was established using the previously reported protocols with some modifications [26, 27]. Briefly, mice were anesthetized with pentobarbital sodium (25 mg/kg, intraperitoneal injection) and then fixed on a stereotaxic device. The scalp was incised along the midline, and the skull was drilled on the right side (0.2 mm anterior and 2.5 mm lateral to the bregma). The tail was sterilized with 70% ethanol and then incised with a sterilized surgical blade. Next, autologous tail arterial blood was collected in a sterilized film and transferred into a 50 *μ*l Hamilton syringe. The needle was inserted at a depth of 3.5 mm into the right ventral basal ganglia. Thirty microliters of autologous blood was injected at a rate of 2 *μ*l/min using a microinjection pump. The needle was slowly withdrawn after retention for 10 minutes. Mice in the sham group underwent identical surgical procedures without blood injection. The core body temperature was maintained at 36.0–36.5°C, as measured using a rectal thermometer, by incubating animals on a heating pad. All animals were allowed to completely recover from anesthesia in a heated chamber before being returned to their home cages.

### 2.4. Cell Culture

The murine BV2 microglial cell line was obtained from the China Center for Type Culture Collection (Wuhan, China) and cultured in Dulbecco's modified Eagle's high glucose medium supplemented with 10% FBS, 100 U/ml penicillin, and 100 *μ*g/ml streptomycin at 37°C and 95% O_2_/5% CO_2_. Twenty-four hours before stimulation, BV2 cells were inoculated into the cell culture plate with serum-free medium. BV2 cells were exposed to 30 *μ*M oxyhemoglobin (OxyHb, Solarbio, Shanghai, China) for 24 hours to mimic ICH, according to the previous studies [15, 28], and the control group was treated with an equivalent volume of phosphate-buffered saline (PBS).

*In vitro* experimental groups were assigned as follows: control group, OxyHb+vehicle group, OxyHb+Omav group, and OxyHb+Omav+ML385 group.

### 2.5. Drug Administration

Omaveloxolone (MedChemExpress, United States) was diluted with 5% dimethyl sulfoxide (DMSO), 5% TWEEN 80, 40% PEG, and 50% sterile saline to a concentration of 1.125 mg/ml for intraperitoneal injection. Intraperitoneal administration (10 mg/kg) of Omav was performed 30 minutes following ICH and once a day over the next two days. The ICH+vehicle group and control group received an equal volume of solvent. For *in vitro* experiments, after 24 hours of culture, 10 nM Omav dissolved in 0.1% DMSO/DMEM was applied with OxyHb to BV2 microglial cells and incubated for another 24 hours. The rational dosage of Omav was based on the previous studies [29–31].

ML385 (MedChemExpress, United States), a selective inhibitor of Nrf2, was dissolved in 5% dimethyl sulfoxide (DMSO), 5% TWEEN 80, 40% PEG, and 50% sterile saline to a concentration of 3.375 mg/ml for *in vivo* experiments. Intraperitoneal administration (10 mg/kg) of ML385 was performed 1 day before ICH modeling and 1 hour before Omav administration over the next few days. Mice in the ICH+vehicle and ICH+Omav groups received an equivalent volume of solvent instead of drug for the same duration as the vehicle control. ML385 was diluted with 0.1% DMSO/DMEM to a concentration of 5 nM for *in vitro* experiments. BV2 cells were pretreated with ML385 2 hours before OxyHb stimulation. The dosage of ML385 was based on the previous studies [32, 33].

### 2.6. Behavioral Assessments

Neurological function was assessed 1 day and 3 days after ICH modeling by investigators who were blinded to the group information. All procedures were performed as previously described [33, 34]. Briefly, for the corner turn test, mice were placed into a corner with a 30-degree angle. The mouse could turn either to the left or the right to leave of the corner, and the percentage of the right turns was calculated after 10 trials. For the forelimb placement test, mice were held by their torsos, and their left side vibrissae were gently brushed on the corner of a countertop. The percentage of trials in which the mouse placed the left forelimb on the edge of the countertop was calculated after 10 trials. For the cylinder test, mice were placed into a transparent cylinder for 5 minutes in 20 trials. The percentage of occasions when the ipsilateral or contralateral forelimb was used to fully contact the wall was recorded, and the percent difference between the ipsilateral and contralateral forelimbs was calculated.

### 2.7. *In Vitro* Phagocytosis Assay

BV2 cells were inoculated on coverslips and stimulated as described above. Erythrocytes were isolated from newly drawn mouse blood with Ficoll (Merck Millipore, Germany). Briefly, the same amounts of PBS and blood were mixed, gently added to Ficoll in a centrifuge tube, and centrifuged at 2000 rpm for 25 minutes with minimum acceleration and deceleration. The erythrocytes were washed twice with PBS before labeling with DiI (Beyotime, China), a fluorescence indicator for the cytomembrane. The labeled erythrocytes were added to the BV2 cells in the plate at a ratio of 10 : 1. After 2 hours of phagocytosis, undigested erythrocytes were dislodged with Red Blood Cell Lysis Buffer (Beyotime, China), and the BV2 cells were labeled with Actin-Tracker Green (Beyotime, China) according to the product manual. The proportion of BV2 cells containing ingested erythrocytes was quantified using fluorescence microscopy. For flow cytometry (FCM), latex beads (Merck Millipore, Germany) with red fluorescence were used for the phagocytosis assay, and the cells were harvested to quantify the phagocytic ability after 2 hours of phagocytosis. The gating strategy of phagocytosis is shown in Sup. Fig 1 B and the data were analyzed using FlowJo software.

### 2.8. Western Blot Analysis

Western blotting was performed as previously described [35]. Protein was extracted from perihematoma samples and BV2 cells with RIPA lysis buffer. Nuclear proteins were extracted according to the instructions of the nuclear and cytoplasmic protein extraction kit (Beyotime, China). Then, equivalent amounts of protein samples were loaded onto sodium dodecyl sulfate–polyacrylamide gels and electrophoresed. The protein was transferred onto PVDF membranes and blocked with 5% nonfat dry milk in buffer, followed by an incubation with primary antibodies against the following proteins overnight at 4°C: *β*-actin (cat. no. ab8226), Nrf2 (cat. no. ab137550), NQO1 (cat. no. ab80588), HO-1 (cat. no. ab52947), and H3 (cat. no. ab1791). The membranes were then washed with Tris-buffered saline containing Tween 20 (TBST) and incubated with appropriate horseradish peroxidase-conjugated secondary antibodies at room temperature for 1 hour. The protein band densities were detected using chemiluminescence with Amersham™ ImageQuant™ 800 (GE Health care, Beijing, China) and quantified using ImageJ software.

### 2.9. Enzyme-Linked Immunosorbent Assay (ELISA)

After appointed treatments, the cell supernatant was harvested and centrifuged at 1,000 rpm for 5 minutes to remove floating cells. The levels of TNF-*α* (cat. no. KE10003) and IL-1*β* (cat. no. KE10003) in the supernatants of treated BV2 cells were examined using ELISA kits (Proteintech) according to the manufacturer's instructions. As for perihematoma tissues, 20 mg tissues was added into 0.2 ml extraction reagent and swayed for 30 minutes at 4°C to gain lysis. After 2 minutes of sonication on ice and 30 minutes of centrifugation at 10,000 × *g*, the concentration of total protein in supernatants was measured. The content of IL-1*β* and TNF-*α* in per mg total protein was quantified according to the standard curve.

### 2.10. Quantitative Real-time PCR

Total RNA was extracted from tissues and cells using TRIzol reagent (Beyotime, China). RNA (1 *μ*g) from each sample was reverse transcribed to cDNAs using a PrimeScript™ RT reagent kit (Takara Bio Inc., Japan) according to the product manuals. Quantitative real-time PCR was performed with SYBR Premix Ex Taq™ (Takara Bio Inc., Japan) on a 7500 Plus Read-Time PCR System. The transcript levels of target genes were normalized to *β*-actin.

### 2.11. ROS Detection

ROS generation was detected using 2′,7′-dichlorodihydrofluorescein diacetate (DCFH-DA, Beyotime, China), which can cross cell membrane and be enzymatically hydrolyzed to DCFH, a fluorogenic indicator of superoxide. Intracellular reactive oxygen species can oxidize nonfluorescent DCFH to generate fluorescent DCF. Briefly, after administering the vehicle, OxyHb, Omav, and ML385 to the appropriate groups, the medium was removed, and the cells were washed with PBS and then stained with 10 nM DCFH-DA for 20 minutes at 37°C in the dark. After 3 washes with PBS and staining nuclei with Hoechst (Beyotime, China), the fluorescence intensities were detected using a microscope (Leica DM6B, Germany) under a fixed exposure condition.

### 2.12. Detection of Mitochondrial Bioactivity

The bioactivity of mitochondria was measured using MitoTracker Red CMXRos (Beyotime, China), which specifically labels active mitochondria in cells. Briefly, CMXRos was added to the cell medium to a final concentration of 50 nM and coincubated for 30 minutes. The cells were then washed with PBS and fixed with 4% paraformaldehyde for 15 minutes. After 3 washes, the cells were harvested for FCM detection using a flow cytometer (Beckman Coulter, United States); the gating strategy of mitochondrial bioactivity detection was shown in Sup. Fig1 A.

### 2.13. Residual Hematoma Volume

The assessment of the residual hematoma volume was based on the previously described methods with some modifications [36]. Briefly, mouse brains were removed after transcardial perfusion with cold PBS and cut into 1 mm thick coronal sections. A set of digital images was acquired and analyzed with ImageJ software to quantify the residual hematoma volume. After converting the image to 8 bits and inverting it, the area of residual hematoma volume was measured, and the difference in gray value between the hematoma and contralateral regions was calculated. The total residual hematoma volume from these sections was then integrated using the following formula *V* = ∑(Areas of hematoma × 1), where *V* is the residual hematoma volume calculated in cubic millimeters. Similarly, the hematoma index was integrated by adding the difference in the gray value.

### 2.14. Hemoglobin Index

The concentration of hemoglobin in the residual hematoma was detected using Drabkin's reagent (Merck Millipore, Germany) according to the manufacturer's instructions. Briefly, the residual hematoma samples were collected and homogenized. After centrifuging the homogenate, the supernatant was mixed with the reagent and incubated for 20 minutes. The absorbance was detected with a microplate reader, and the concentration of hemoglobin was calculated from a standard curve.

### 2.15. Immunofluorescence Staining

Immunofluorescence staining was performed as previously described [37]. Mice were sacrificed and perfused with cold PBS followed by 4% cold paraformaldehyde (PFA). The brains were removed intact, immersed in 4% PFA, and incubated at 4°C for 24 hours. After dehydration in a 30% sucrose solution, the samples were sliced into 8 *μ*m coronal sections for staining. Brain slices were then sequentially washed with PBS and blocked with 10% donkey serum and 0.3% Triton X-100 to prevent nonspecific binding. The primary antibodies used for immunofluorescence staining were as follows: Iba-1 (cat. no. ab5076), iNOS (cat. no. ab178945), and Arg1 (cat. no. 16001-1-AP). After an overnight incubation at 4°C and washing, slices were incubated with secondary antibodies for 2 hours at room temperature in the dark. The sections were rinsed and mounted using Antifade Mounting Medium with DAPI (cat. no. MA0222, Meilunbio). The images were captured with a fluorescence microscope.

### 2.16. Statistical Analysis

All data are presented as the means ± standard errors. All statistical analyses were performed using SPSS 26.0 software. Comparisons between two groups were evaluated using unpaired Student's *t* tests. Differences between different treatment groups were analyzed using one-way ANOVA with Bonferroni's post hoc multiple comparison tests. Statistical significance was defined as *P* < 0.05.

## 3. Results

### 3.1. Omav Increased Nrf2, NQO1, and HO-1 Expressions and Nrf2 Nuclear Translocation in OxyHb-Stimulated BV2 Microglial Cells

**Figure 1:**
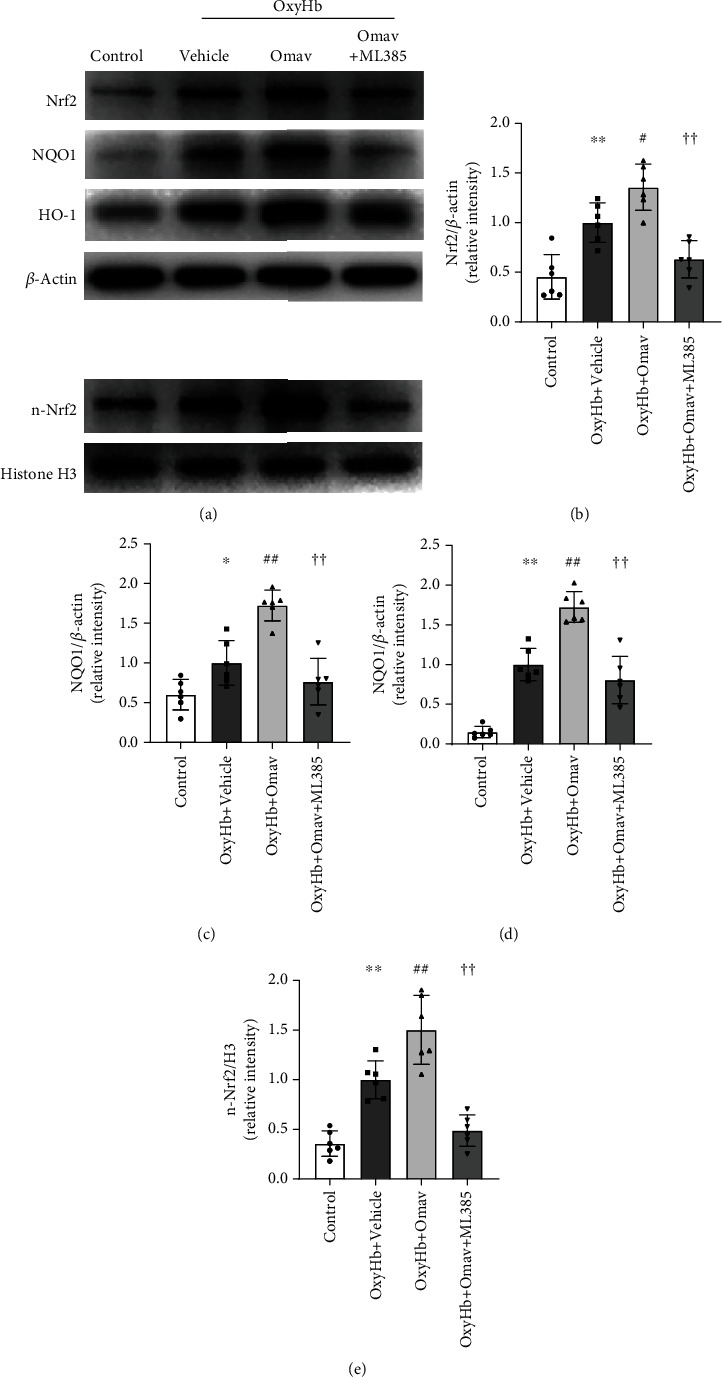
Omav increased Nrf2, NQO1, and HO-1 expressions and Nrf2 nuclear translocation *in vitro*. (a) Western blots showing the levels of Nrf2, NQO1, HO-1, and n-Nrf2 in BV2 cells treated with vehicle, OxyHb, Omav, and ML385. (b–e) Relative quantification of Nrf2, NQO1, HO-1, and n-Nrf2 expression (*n* = 6). Data are presented as the means ± SEM. ^∗^*P* < 0.05 and ^∗∗^*P* < 0.01 compared with the control group; ^#^*P* <0.05 and ^##^*P* < 0.01 compared with the OxyHb group; ^††^*P* < 0.01 compared with the OxyHb+Omav group.

First, we explored the effect of Omav on microglia *in vitro* by measuring the expression of Nrf2 and its downstream proteins in the BV2 cell line. The western blot results indicated that Omav increased Nrf2 expression in cells under OxyHb stress, and this effect was partially blocked by ML385, a selective inhibitor of Nrf2 (Figures 1(a) and 1(b) and Sup. Fig. 2 a, b). Consistent with Nrf2, the expression of HO-1 and NQO1 was increased in the OxyHb+Omav group compared to the OxyHb group, and ML385 also reversed this change (Figures 1(c) and 1(d)). Since the nuclear translocation of Nrf2 primes downstream protein expression, we additionally measured nuclear Nrf2 (n-Nrf2) and cytoplasmic Nrf2 levels in each group. As shown in Figure 1(e) and Sup. Fig. 2 c, d, Omav increased n-Nrf2 and cyto-Nrf2 levels, while ML385 treatment diminished n-Nrf2 and cyto-Nrf2 levels. Thus, Omav promotes the expression of antioxidant proteins in BV2 cells by facilitating the expression and translocation of Nrf2.

### 3.2. Omav Decreased M1 Microglial-Related Gene Expression but Increased M2 Microglial-Related Gene Expression *In Vitro*

**Figure 2:**
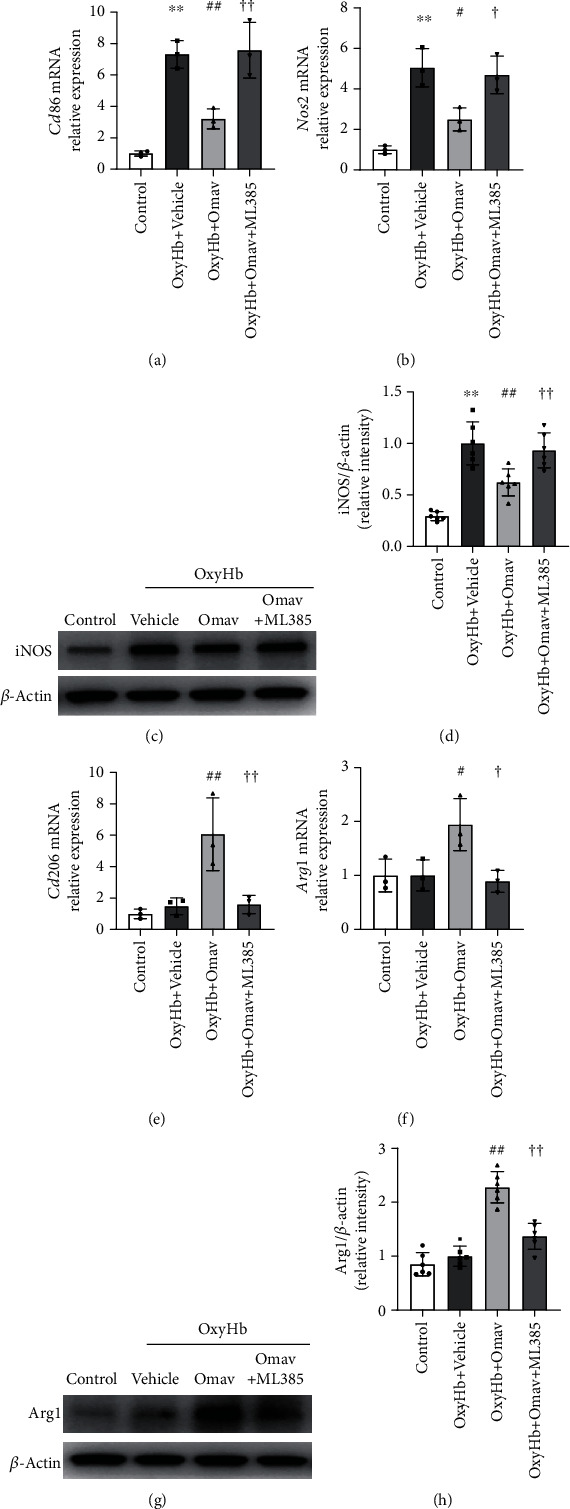
Omav reduced the expression of M1-related genes and proteins but increased the expression of M2-related genes and proteins *in vitro*. (a and b) Levels of the *Cd86* and *Nos2* mRNAs in the control group, OxyHb group, OxyHb+Omav group, and OxyHb+Omav+ML385 group (*n* = 3). (c and d) Representative western blots bands and the quantification of the expression of iNOS (*n* = 6). (e and f) mRNA Levels of the *Cd206* and *Arg1* assessed via qPCR (*n* = 3). (g and h) Representative western blots bands and the quantification of the expression of Arg1 (*n* = 6). Data are presented as the means ± SEM. ^∗∗^*P* < 0.01 compared with the control group; ^#^*P* < 0.05 and ^##^*P* < 0.01 compared with the OxyHb group; ^†^*P* < 0.05 and ^††^*P* < 0.01 compared with the OxyHb+Omav group.

According to the previous studies, OxyHb stimulation caused microglia to activate the M1 phenotype [38]. We assessed the expression of phenotype-related genes and proteins to explore the effect of Omav on microglial polarization. BV2 microglial cells were treated as described in Section 3.1 for 6 hours. qRT-PCR revealed that Omav inhibited M1-related gene (*Cd86* and *Nos2*) transcription (Figures 2(a) and 2(b)). Western blots of iNOS showed a consistent tendency (Figures 2(c) and 2(d)). Meanwhile, M2-related gene (*Cd206* and *Arg1*) transcription and Arg1 expression were promoted by Omav (Figures 2(e)–2(h)). Pretreatment with ML385 blocked the effect of Omav. Taken together, these results indicated that Omav modulated microglial polarization toward the M2 phenotype following OxyHb stimulation.

### 3.3. Omav Administration Decreased the Proinflammatory Cytokine Production but Promoted Phagocytosis after OxyHb Stimulation in BV2 Microglial Cells

**Figure 3:**
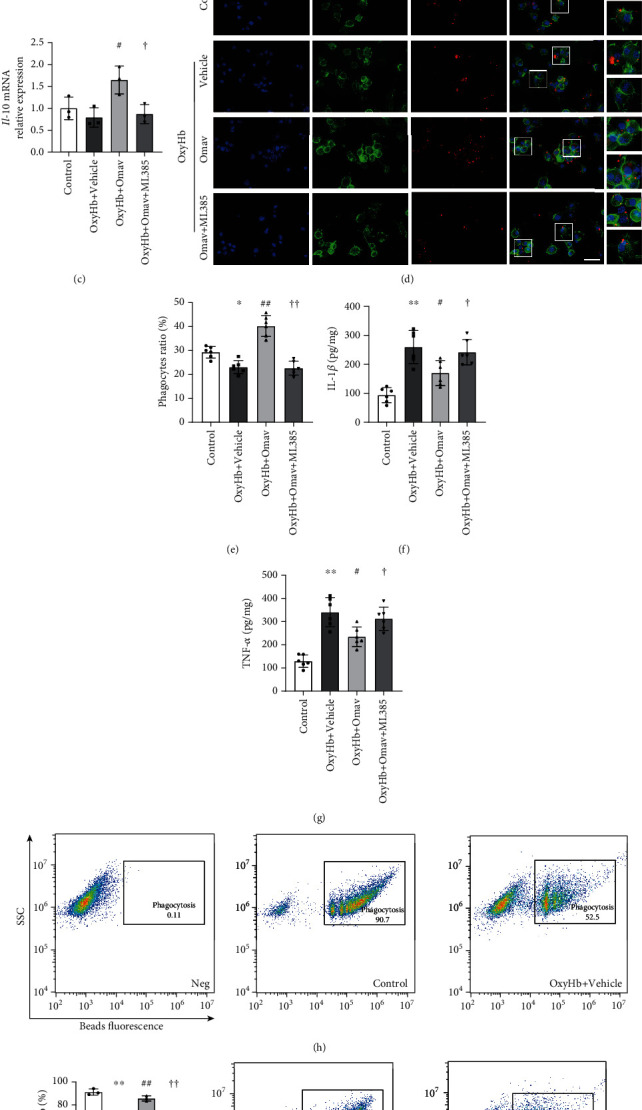
Omav reduced the expression of inflammatory cytokines but enhanced BV2 phagocytosis under the stress of OxyHb. (a–c) Levels of the *Il-1β, Tnf-α*, and *Il-10* mRNAs in treated BV2 cells (*n* = 3). (d) Representative fluorescent images of the phagocytosis assay in BV2 cells. *β*-Actin in BV2 cells was labeled with phalloidin-FITC, and RBCs were labeled with DiI. Scale bar: 50 *μ*m. (e) Quantification of the phagocyte proportion according to fluorescence (*n* = 6). (f and g) The concentrations of IL-1*β* and TNF-*α* were detected using ELISAs (*n* = 6). (h) Phagocytosis ratio of BV2 cells in each group detected via FCM. (i) Quantification of the ratio of BV2 cells phagocytoses labeled RBCs from each group (*n* = 3). Data are presented as the means ± SEM. ^∗^*P* < 0.05 and ^∗∗^*P* < 0.01 compared with the control group; ^#^*P* < 0.05 and ^##^*P* < 0.01 compared with the OxyHb group; ^†^*P* < 0.05 and ^††^*P* < 0.01 compared with the OxyHb+Omav group.

We tested the proinflammatory or prophagocytic functions of BV2 cells treated with Omav to further verify the pharmacological effects of Omav on the microglial polarization. The mRNA expression of inflammatory cytokines (*Il-1β* and *Tnf-α*) was upregulated upon stimulation with OxyHb but downregulated after treatment with Omav (Figures 3(a) and 3(b)). Regarding the M2 phenotype, Omav increased the mRNA expression of *Il-10*, an anti-inflammatory M2 signature cytokine secreted by M2 microglia (Figure 3(c)). Fluorescence imaging showed that Omav improved the phagocyte ratio of BV2 cells (Figures 3(d) and 3(e)), which was decreased by pretreatment with ML385. We further detected the secretion of IL-1*β* and TNF-*α* using ELISAs, which showed that Omav decreased the levels of these cytokines compared with the OxyHb treatment group (Figures 3(f) and 3(g)). Both transcriptional and translational changes induced by Omav were blocked by ML385 pretreatment. Furthermore, flow cytometry (FCM) revealed a parallel result of higher phagocytosis of latex beads by the OxyHb+Omav group than by the OxyHb+vehicle group and OxyHb+Omav+ML385 group (Figures 3(h) and 3(i)). Collectively, these results suggested that Omav treatment inhibited M1-like polarization but promoted M2-like phenotype activation following OxyHb challenge.

### 3.4. Omav Reduced ROS Production and Increased Mitochondrial Membrane Potential after OxyHb *In Vitro*

**Figure 4:**
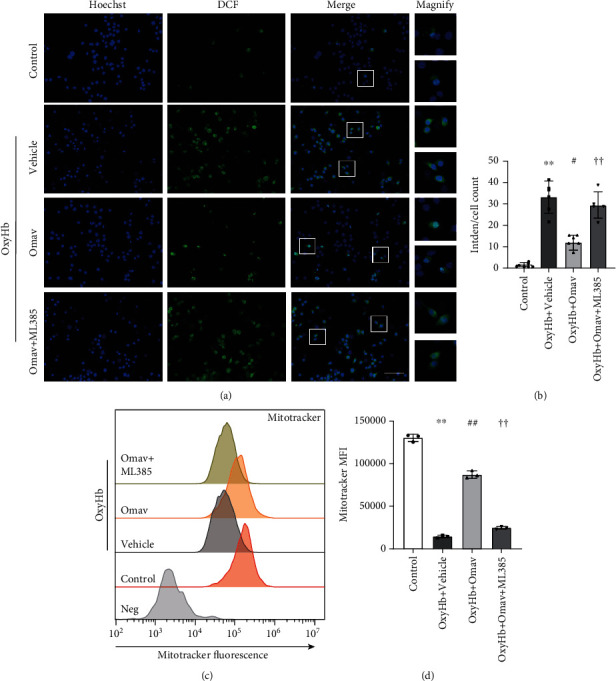
Omav reduced ROS generation and increased mitochondrial membrane potential of BV2 in response to OxyHb. (a) ROS levels were detected using an ROS assay kit with fluorescence microscopy after exposure to the corresponding stimulus; scale bar: 50 *μ*m. (b) Quantification of fluorescence intensity per cell in each group (*n* = 6). (c) Mitochondrial bioactivity was detected using MitoTracker Red CMXRos with FCM. (d) Quantification of the mean fluorescence intensity (MFI) assessed via FCM (*n* = 3). Data are presented as the means ± SEM. ^∗^*P* < 0.05 and ^∗∗^*P* < 0.01 compared with the control group; ^#^*P* < 0.05 and ^##^*P* < 0.01 compared with the OxyHb group; ^†^*P* < 0.05 and ^††^*P* < 0.01 compared with the OxyHb+Omav group.

Intracellular ROS induced a self-propelling cycle to maintain the continued activation of the M1-like phenotype [39]. We hypothesized that the mechanisms underlying the function of Omav in modulating microglial polarization are associated with ROS. Fluorescence imaging showed that OxyHb increased the mean ROS level, and Omav treatment significantly reduced the elevated fluorescence signal, while the ML385 pretreatment attenuated the inhibitory effect of Omav on OxyHb-induced ROS generation (Figures 4(a) and 4(b)). We then performed FCM to detect the mitochondrial membrane potential, which indicates the bioactivity of mitochondria. Compared with the control group, OxyHb stimulation markedly decreased mitochondrial bioactivity, Omav administration attenuated the impairment caused by OxyHb, and the protective effect of Omav on mitochondrial bioactivity was blocked by the ML385 pretreatment (Figures 4(c) and 4(d)). These results indicated that the modulation of microglial polarization by Omav was associated with reducing ROS generation and mitochondrial protection.

### 3.5. Omav Downregulated Proinflammatory Cytokines after ICH

**Figure 5:**
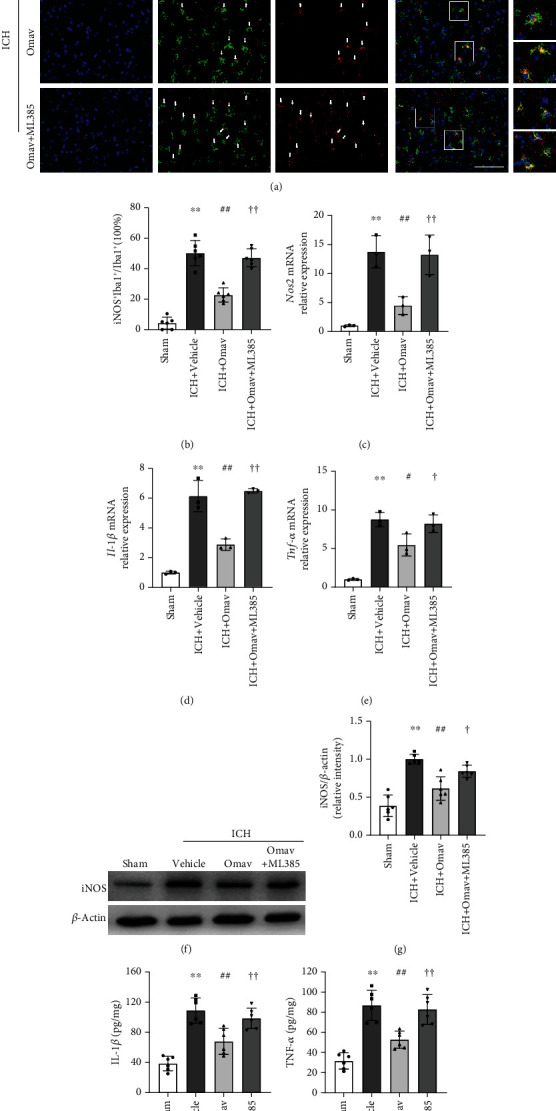
Omav reduced the expression of proinflammatory genes and proteins after ICH. (a) Immunofluorescence staining for Iba-1 (green) and iNOS (red) in the ipsilateral basal ganglia region 3 days after ICH; the nuclei were stained with DAPI (blue); scale bar: 50 *μ*m (*n* = 6). (b) Quantification of the ratio of microglia expressing iNOS. (c–e) qRT-PCR analysis of the expression of the mRNAs encoding iNOS, IL-1*β*, and TNF-*α* (*n* = 3). (f) Western blot showing iNOS levels in peripheral hematoma tissues from each group (*n* = 6). (g) Quantitative analysis of the iNOS band. (h–i) The concentrations of IL-1*β* and TNF-*α* in perihematoma 3 days after ICH were detected using ELISAs (*n* = 6). Data are presented as the means ± SEM. ^∗^*P* < 0.05 and ^∗∗^*P* < 0.01 compared with the control group; ^#^*P* < 0.05 and ^##^*P* < 0.01 compared with the OxyHb group; ^†^*P* < 0.05 and ^††^*P* < 0.01 compared with the OxyHb+Omav group.

After documenting the modulatory effect of Omav on BV2 microglial polarization, we assumed that Omav exerted anti-inflammatory effects on the ICH insult. Inducible nitric oxide synthase (iNOS) is a key factor associated with microglia-mediated neuropathology [40]. Immunofluorescence staining revealed that Omav decreased the ratio of iNOS-positive microglia around the perihematomal region 1 and 3 days after ICH (Figures 5(a) and 5(b); Sup. Fig. 3A, B; and Sup. Fig. 4A, C). We also discovered diminished expression of the iNOS (*Nos2*), IL-1*β*, and TNF-*α* mRNAs (Figures 5(c), 5(d), and 5(e)) in the ICH+Omav group. Western blot showed that iNOS was expressed at lower levels in the ICH+Omav group than in the ICH+vehicle group (Figures 5(f) and 5(g)). In addition, the results of ELISA suggested that Omav treatment significantly reduced the levels of IL-1*β* and TNF-*α* at 1 and 3 days after ICH (Figures 5(h) and 5(I) and Sup. Fig. 4E, F). Furthermore, with ML385 pretreatment, the percentage of iNOS-positive cells in brain sections was increased, and the expression levels of M1 microglial markers were increased. Together, these findings suggested that Omav exerted an anti-inflammatory effect that attenuated M1 microglial activation after ICH injury *in vivo*.

### 3.6. Omav Increased the Expression of Arg1 on Microglia and Promoted Hematoma Resolution 3 Days Post-ICH

**Figure 6:**
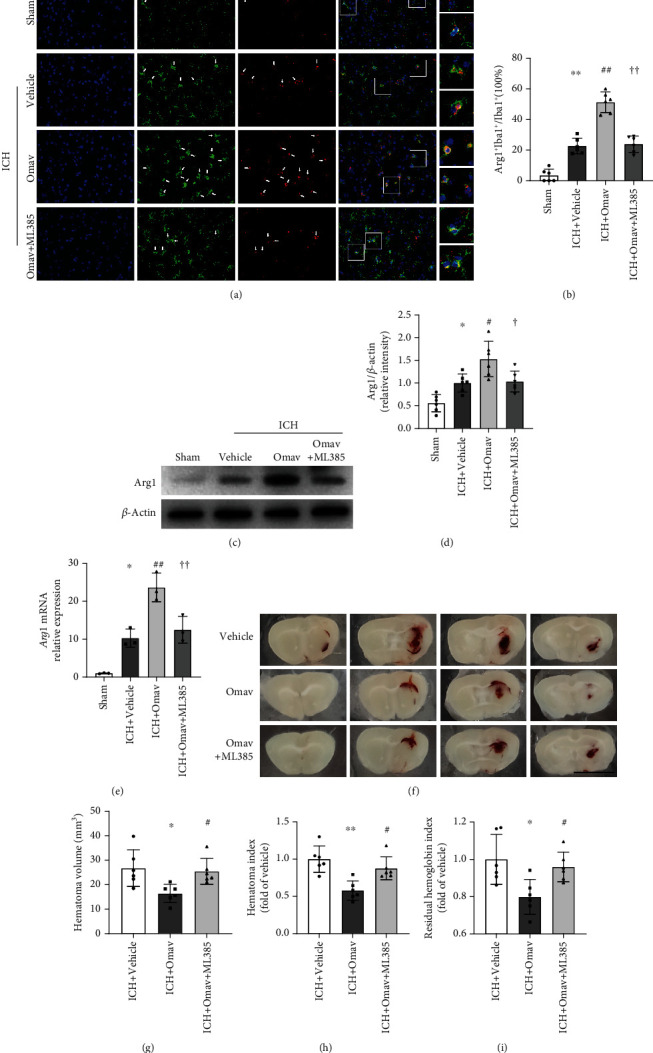
Omav promoted M2-like microglial polarization and accelerated hematoma resolution after ICH. (a) Immunofluorescence staining for Iba-1 (green) and Arg1 (red) in the ipsilateral basal ganglia region; the nuclei were stained with DAPI (blue); scale bar: 50 *μ*m (*n* = 6). (b) Quantification of the ratio of microglia expressing Arg1. (c) Western blot showing Arg1 levels in peripheral hematoma tissues from each group (*n* = 6). (d) Quantitative analysis of the Arg1 band. (e) qRT-PCR analysis of the expression of the Arg1 mRNA (*n* = 3). (f) Representative images of brain slices from each group; scale bar: 5 mm. (g) Quantification of the hematoma volume in each group (*n* = 6). (h) Quantification of the hematoma index (hematoma volume × density), which considers incomplete hematoma absorption (*n* = 6). (a) Quantification of the residual hemoglobin content detected using Drabkin's reagent (*n* = 6). Data are presented as the means ± SEM. ^∗^*P* < 0.05 and ^∗∗^*P* < 0.01 compared with the control group; ^#^*P* < 0.05 and ^##^*P* < 0.01 compared with the OxyHb group; ^†^*P* < 0.05 and ^††^*P* < 0.01 compared with the OxyHb+Omav group.

Generally, M2 polarization follows M1 activation at later stages in ICH pathology, and Arg1 is a crucial M2 marker that downregulates iNOS activity [16]. Here, immunofluorescence staining revealed that Omav increased the percentage of Arg1+Iba1+/Iba1+ cells around the perihematomal region (Figures 6(a) and 6(b); Sup. Fig. 3c, d; and Sup. Fig. 4b, d). Western blot analysis revealed an increase in Arg1 protein levels in the ICH+Omav group compared to the ICH+vehicle group (Figures 6(c) and 6(d)). qRT-PCR showed increased expression of the *Arg1* mRNA following Omav treatment (Figure 6(e)). Furthermore, we acquired images of 1 mm thick coronal sections to evaluate the residual hematoma volume before collecting the hematoma for hemoglobin tests and protein samples for western blotting (Figure 6(f)). Measurement of the area and gray value of these images showed a lower hematoma volume and hematoma index in the ICH+Omav group than in the other groups (Figures 6(g) and 6(h)). Consistent with the volume changes, the residual hemoglobin content was decreased by Omav treatment (Figure 6(i)). Collectively, Omav enhanced M2 microglial polarization to protect against ICH, thereby promoting hematoma absorption.

### 3.7. Omav Treatment Alleviated Neurological Deficits in Mouse ICH Models

**Figure 7:**
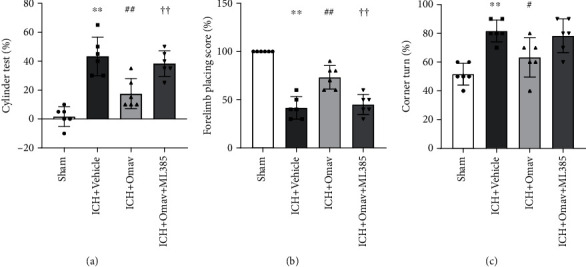
Omav alleviated neurological deficits in mice 3 days after ICH. The results of the following neurological behavior tests: the cylinder test (a), forelimb placement score (b), and corner test (c) (*n* = 6). ^∗∗^*P* < 0.01 compared with the control group; ^#^*P* < 0.05 and ^##^*P* < 0.01 compared with the OxyHb group; ^††^*P* < 0.01 compared with the OxyHb+Omav group.

We assessed the neurological functions of each group 3 days after ICH modeling to intuitively evaluate the neuroprotective effect of Omav. The results of the cylinder test showed that Omav decreased the ratio of using the nonimpaired forelimb for weight shifting movements after ICH (Figure 7(a)). In addition, the administration of Omav increased the forelimb placement score (Figure 7(b)). Moreover, the corner turn test showed a lower ratio of turning right in the ICH+Omav group (Figure 7(c)). Based on these results, Omav administration improved the sensorimotor/proprioceptive capacity 3 days after ICH. In contrast, inhibition of Nrf2 by ML385 blocked the amelioration of neurological deficits. Taken together, the modulation of microglial polarization by Omav might be a functional neuroprotective target after ICH.

## 4. Discussion

This study examined the effect of Omav, a potent Nrf2 activator, on ICH both *in vivo* and *in vitro*. Omav attenuated ICH-induced neuroinflammation and promoted hematoma clearance by modulating microglial M1/M2 polarization, subsequently facilitating neurological recovery after ICH. Omav promoted the nuclear translocation of Nrf2 and increased the expression of HO-1 and NQO1 to then reduce ROS accumulation and preserve mitochondrial functions in microglia. In addition, ML385-mediated inhibition of Nrf2 abolished the protective effects of Omav on ICH.

Hematoma evacuation was once the mainstay of ICH treatment, yet the improvement of functional outcome or mortality was limited, as reported in several well-known clinical trials, such as STICH and MISTIE [41, 42]. These results do not deny the role of early clearance of hematoma but rather highlight that secondary brain injury is as important as primary injury in influencing long-term neurological recovery after ICH. As key innate immune cells, microglia are the first nonneuronal cells to respond to acute brain injuries [43–45]. M1 microglia are presumed to be proinflammatory and release cytokines such as TNF-*α*, IL-1*β*, and IL-6, whereas M2 microglia are presumed to secrete anti-inflammatory cytokines such as IL-10 and contribute to the phagocytosis of hematoma [12]. Previous studies have revealed dynamic changes in the M1 to M2 phenotype over time in response to traumatic brain injury [46], ischemic brain injury [10], and ICH. In a study of ICH in mice, microglia responded to hemorrhagic attack within the first hour after surgery and presented an M1-like phenotype. Markers of M2 microglia increased later than those of M1 microglia, peaked from days 3 to 7 after ICH induction. In clinical studies, both M1 and M2 microglia were detected in the hematoma of patients with ICH within 30 hours after ICH onset. More importantly, higher levels of IL-10 in the hematoma and serum and lower amounts of M1 microglia were independently associated with a better recovery at 90 days after ICH. In this regard, modulating the phenotypic changes of activated microglia may provide opportunities for future therapeutic interventions. Consistent with previous studies, our study indicated that both M1 and M2 microglia existed after ICH induction in mice. The proportions of iNOS-positive and Arg-positive microglia cells in ICH groups at 3 days were higher than those in the corresponding group at 1 day post-ICH. However, the proportion of iNOS-positive microglia in the Omav treatment group was lower than that in the vehicle group, and the ratio of Arg1-positive microglia was higher than that in the vehicle group. This indicated that Omav administration may advance the polarization of microglia toward the M2 phenotype, which ultimately attenuated the neurological deficits of ICH mice. Indeed, the M1/M2 paradigm is oversimplified to describe the true nature of microglia, especially in complicated situations such as ICH. Here in this study, we tend to use the concept of M1 and M2 to represent the functional properties of microglia rather than emphasize the polarization itself. Fortunately, as the methodology advances, the identification and classification of microglia are becoming more adequate and accurate. For example, the discovery of disease-associated microglia (DAM) has vastly improved our understanding of the diversity and functional role of microglia in AD. Further investigations are urgently needed to better characterize microglia in other brain diseases, including ICH.

In microglia, the main source of ROS is the activation of NADPH oxidase (NOX) and nitric oxide synthase (NOS) enzymes [47, 48]. ROS and the subsequently formed peroxynitrite contribute to cell death by damaging mitochondria and DNA, inhibiting the activity of mitochondrial complexes and the production of ATP, and promoting lipid peroxidation [49, 50]. In addition, ROS production and redox homeostasis have been shown to affect the phenotype of microglia. Pharmacological inhibition of NOX or knockout of its functional subunit p47phox shift LPS-challenged microglia from the M1 phenotype to the M2 phenotype [51]. A mitochondria-targeted antioxidant alleviated brain damage in an autologous blood injection-induced ICH model by polarizing microglia to the M2 phenotype [11]. According to these studies, modulating oxidative stress may be a potential therapeutic target for treating ICH in the future. Antioxidant drugs such as edaravone have been shown to scavenge ROS and modulate the phenotype of microglia either in individuals with hemorrhagic stroke or LPS-induced neuroinflammation [52, 53], yet they failed to reduce mortality and provide long-term clinical benefits in clinical studies [54, 55]. Thus, more efforts are needed to develop novel antioxidant therapies that improve the outcomes of patients with ICH.

Nrf2 regulates a variety of antioxidant genes and is tightly related to redox hemostasis [56]. Nrf2-/- mice suffered more severe brain damage and had a significantly lower survival rate than wild-type mice after ICH [19, 57]. Deletion of Nrf2 impaired hematoma clearance and enhanced neuroinflammation after ICH, while the induction of HO-1, one of the major targets of Nrf2, polarizes microglia/macrophages to the M2 phenotype [57, 58]. Omav is one of the most potent activators of Nrf2. Oral administration of Omav to monkeys is associated with dose-proportional concentrations in the brain and induces Nrf2 target gene mRNA expression [59]. More importantly, Omav is currently being investigated in clinical trials as a treatment for mitochondrial myopathies (NCT02255422), melanoma (NCT02259231), non-small-cell lung cancer (NCT02029729), and Friedreich ataxia (FA, NCT02255435). The results of FA research revealed that Omav is safe and well tolerated, and Omav improved the neurological functions of patients with FA. In this study, we found that Omav alleviated ROS accumulation in microglia by activating Nrf2. Furthermore, Omav downregulates iNOS-positive microglia polarization but promotes Arg1-positive phenotype, which led to the attenuation of ICH-induced neuroinflammation and the acceleration of hematoma clearance. Based on these data, Omav might represent a promising candidate for treating ICH in the future.

## Data Availability

The raw data involved in the manuscript are available on reasonable request.

## Abbreviations

ICH: Intracerebral hemorrhage
Nrf2: Nuclear factor erythroid-2 related factor 2
Omav: Omaveloxolone
HO-1: Heme oxygenase-1
NQO1: NAD(P)H: quinone oxidoreductase-1
ROS: Reactive oxygen species
DMSO: Dimethyl sulfoxide
PBS: Phosphate-buffered saline
qRT-PCR: Quantitative Real-time polymerase chain reaction
OxyHb: Oxyhemoglobin
SOD: Superoxide dismutase
CNS: Central nervous system
DCFH-DA: 2′,7′-Dichlorodihydrofluorescein diacetate
FCM: Flow cytometry
PFA: Paraformaldehyde.

## Supplementary Materials

Supplementary Materials Supplementary Figure 1: (a) gating strategy for FCM in mitochondrial bioactivity detection. (b) In vitro phagocytosis gating strategy. Supplementary Figure 2: (a) western blots showing the levels of Nrf2 in BV2 cells treated with vehicle and ML385 under the stress of OxyHb. (b) Quantification of the Nrf2 expression (*n* = 6). (c) Western blots showing the levels of cytoplasmic Nrf2 in BV2 cells treated with vehicle, OxyHb, Omav, and ML385. (d) Quantification of the cyto-Nrf2 expression (*n* = 6). Data are presented as the means ± SEM. ^∗^*P* < 0.05 and ^∗∗^*P* < 0.01 compared with the control group; ^#^*P* < 0.05 and ^##^*P* < 0.01 compared with the OxyHb group; ^††^*P* < 0.01 compared with the OxyHb+Omav group. Supplementary Figure 3: (A) quantification of the number of cells expressing iNOS. (b) Quantification of number of Iba1-positive cells (*n* = 6). (c) Quantification of the count of cells expressing Arg1. (d) Quantification of count of Iba1+ cells (*n* = 6). Data are presented as the means ± SEM. ^∗^*P* < 0.05 and ^∗∗^*P* < 0.01 compared with the Control group; *P* < 0.05 and *P* < 0.01 compared with the OxyHb group; ^††^*P* < 0.01 compared with the OxyHb+Omav group. Supplementary Figure 4: (A) immunofluorescence staining for Iba-1 (green) and iNOS (red) in the ipsilateral basal ganglia region 1 day after ICH; the nuclei were stained with DAPI (blue); scale bar: 50 *μ*m. (B) Immunofluorescence staining for Iba-1 (green) and Arg1 (red) in the ipsilateral basal ganglia region 1 day after ICH; the nuclei were stained with DAPI (blue); scale bar: 50 *μ*m. (c) Quantification of the ratio of microglia expressing iNOS. (d) Quantification of the ratio of microglia expressing Arg1. (E–F) The concentrations of IL-1*β* and TNF-*α* in perihematoma 1 day after ICH were detected using ELISAs (*n* = 6). Data are presented as the means ± SEM. ^∗^*P* < 0.05 and ^∗∗^*P* < 0.01 compared with the control group; ^##^*P* < 0.01 compared with the OxyHb group; ^†^*P* < 0.05 and ^††^*P* < 0.01 compared with the OxyHb+Omav group.
