# Supplementary figures and images for: The Novel Nrf2 Activator Omaveloxolone Regulates Microglia Phenotype and Ameliorates Secondary Brain Injury after Intracerebral Hemorrhage in Mice
Source: Oxid Med Cell Longev. 2022 Mar 11;2022:4564471. doi: 10.1155/2022/4564471 (PMC8933082; doi:10.1155/2022/4564471)

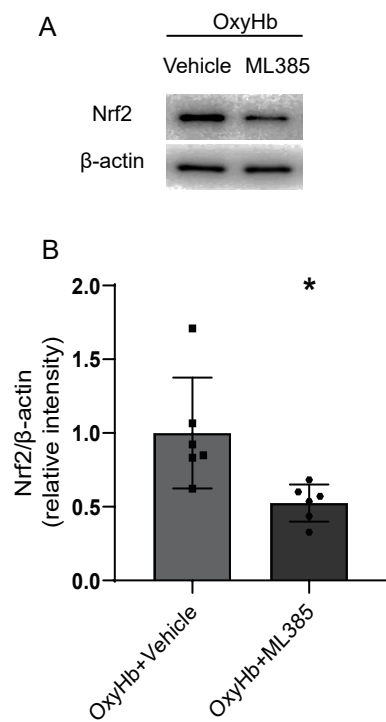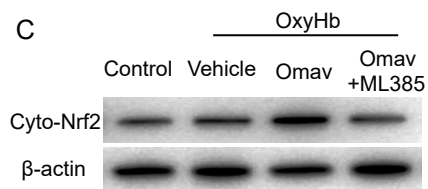

Supplement: Supplementary Materials — Supplementary Figure 1: (a) gating strategy for FCM in mitochondrial bioactivity detection. (b) In vitro phagocytosis gating strategy. Supplementary Figure 2: (a) western blots showing the levels of Nrf2 in BV2 cells treated with vehicle and ML385 under the stress of OxyHb. (b) Quantification of the Nrf2 expression (n = 6). (c) Western blots showing the levels of cytoplasmic Nrf2 in BV2 cells treated with vehicle, OxyHb, Omav, and ML385. (d) Quantification of the cyto-Nrf2 expression (n = 6). Data are presented as the means ± SEM. ∗P < 0.05 and ∗∗P < 0.01 compared with the control group; #P < 0.05 and ##P < 0.01 compared with the OxyHb group; ††P < 0.01 compared with the OxyHb+Omav group. Supplementary Figure 3: (A) quantification of the number of cells expressing iNOS. (b) Quantification of number of Iba1-positive cells (n = 6). (c) Quantification of the count of cells expressing Arg1. (d) Quantification of count of Iba1+ cells (n = 6). Data are presented as the means ± SEM. ∗P < 0.05 and ∗∗P < 0.01 compared with the Control group; P < 0.05 and P < 0.01 compared with the OxyHb group; ††P < 0.01 compared with the OxyHb+Omav group. Supplementary Figure 4: (A) immunofluorescence staining for Iba-1 (green) and iNOS (red) in the ipsilateral basal ganglia region 1 day after ICH; the nuclei were stained with DAPI (blue); scale bar: 50 μm. (B) Immunofluorescence staining for Iba-1 (green) and Arg1 (red) in the ipsilateral basal ganglia region 1 day after ICH; the nuclei were stained with DAPI (blue); scale bar: 50 μm. (c) Quantification of the ratio of microglia expressing iNOS. (d) Quantification of the ratio of microglia expressing Arg1. (E–F) The concentrations of IL-1β and TNF-α in perihematoma 1 day after ICH were detected using ELISAs (n = 6). Data are presented as the means ± SEM. ∗P < 0.05 and ∗∗P < 0.01 compared with the control group; ##P < 0.01 compared with the OxyHb group; †P < 0.05 and ††P < 0.01 compared with the OxyHb+Omav group. [file 4564471.f1.zip › Supplemental Figure 2.pdf]

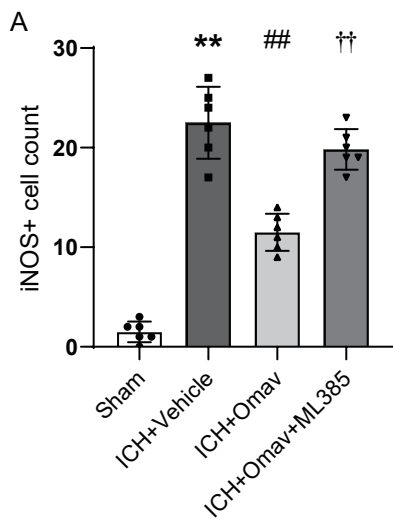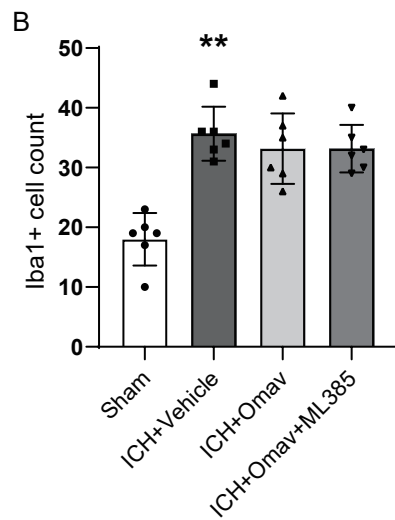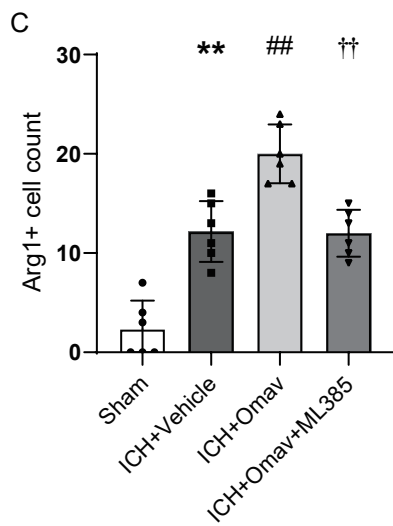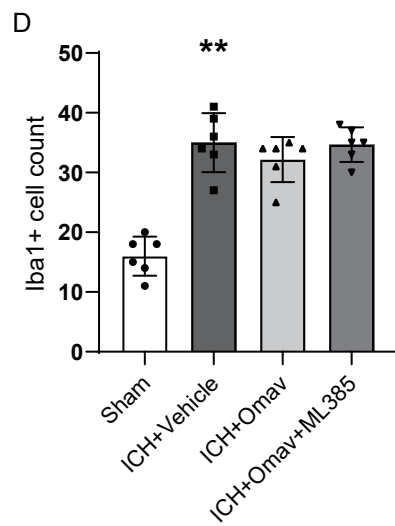

Supplement: Supplementary Materials — Supplementary Figure 1: (a) gating strategy for FCM in mitochondrial bioactivity detection. (b) In vitro phagocytosis gating strategy. Supplementary Figure 2: (a) western blots showing the levels of Nrf2 in BV2 cells treated with vehicle and ML385 under the stress of OxyHb. (b) Quantification of the Nrf2 expression (n = 6). (c) Western blots showing the levels of cytoplasmic Nrf2 in BV2 cells treated with vehicle, OxyHb, Omav, and ML385. (d) Quantification of the cyto-Nrf2 expression (n = 6). Data are presented as the means ± SEM. ∗P < 0.05 and ∗∗P < 0.01 compared with the control group; #P < 0.05 and ##P < 0.01 compared with the OxyHb group; ††P < 0.01 compared with the OxyHb+Omav group. Supplementary Figure 3: (A) quantification of the number of cells expressing iNOS. (b) Quantification of number of Iba1-positive cells (n = 6). (c) Quantification of the count of cells expressing Arg1. (d) Quantification of count of Iba1+ cells (n = 6). Data are presented as the means ± SEM. ∗P < 0.05 and ∗∗P < 0.01 compared with the Control group; P < 0.05 and P < 0.01 compared with the OxyHb group; ††P < 0.01 compared with the OxyHb+Omav group. Supplementary Figure 4: (A) immunofluorescence staining for Iba-1 (green) and iNOS (red) in the ipsilateral basal ganglia region 1 day after ICH; the nuclei were stained with DAPI (blue); scale bar: 50 μm. (B) Immunofluorescence staining for Iba-1 (green) and Arg1 (red) in the ipsilateral basal ganglia region 1 day after ICH; the nuclei were stained with DAPI (blue); scale bar: 50 μm. (c) Quantification of the ratio of microglia expressing iNOS. (d) Quantification of the ratio of microglia expressing Arg1. (E–F) The concentrations of IL-1β and TNF-α in perihematoma 1 day after ICH were detected using ELISAs (n = 6). Data are presented as the means ± SEM. ∗P < 0.05 and ∗∗P < 0.01 compared with the control group; ##P < 0.01 compared with the OxyHb group; †P < 0.05 and ††P < 0.01 compared with the OxyHb+Omav group. [file 4564471.f1.zip › Supplemental Figure 3.pdf]

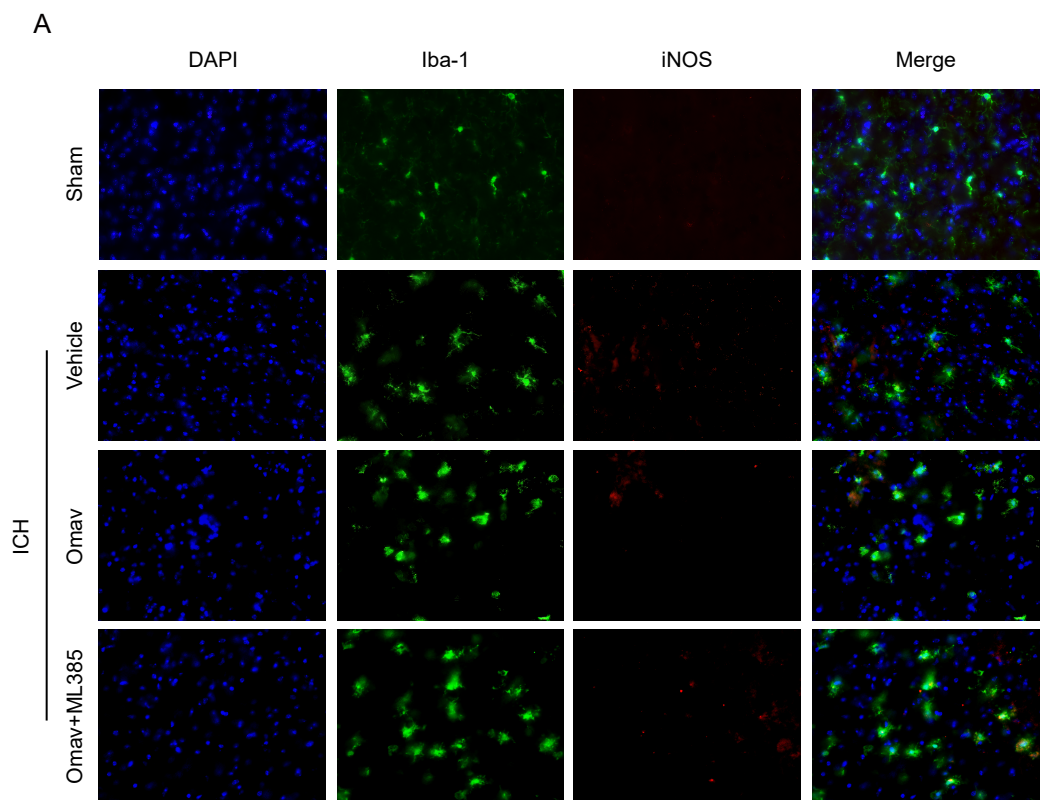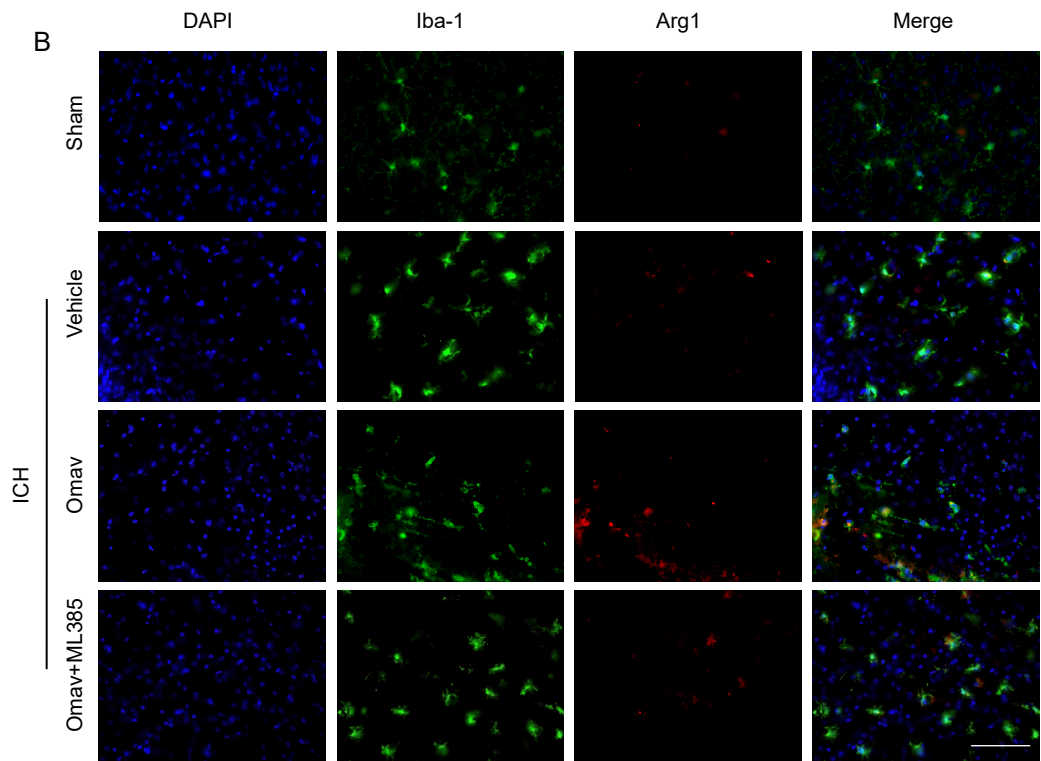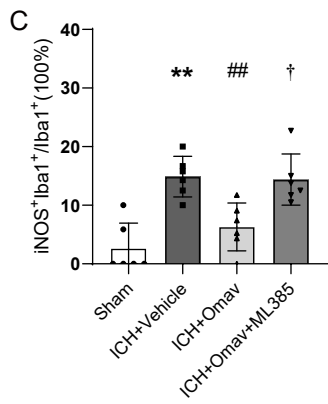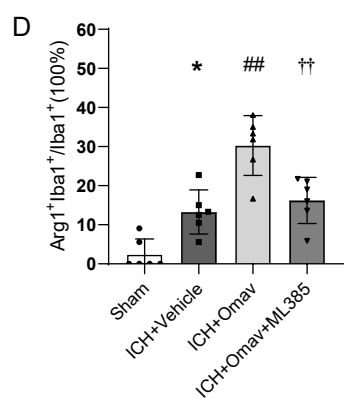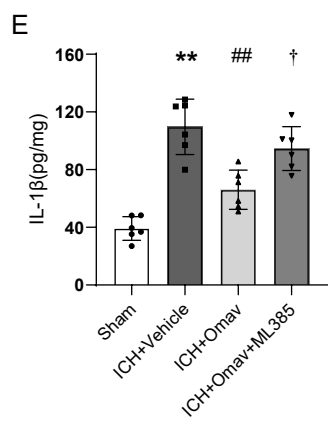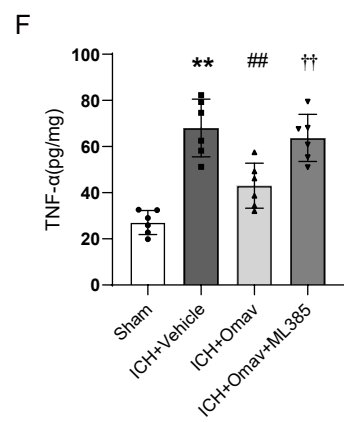

Supplement: Supplementary Materials — Supplementary Figure 1: (a) gating strategy for FCM in mitochondrial bioactivity detection. (b) In vitro phagocytosis gating strategy. Supplementary Figure 2: (a) western blots showing the levels of Nrf2 in BV2 cells treated with vehicle and ML385 under the stress of OxyHb. (b) Quantification of the Nrf2 expression (n = 6). (c) Western blots showing the levels of cytoplasmic Nrf2 in BV2 cells treated with vehicle, OxyHb, Omav, and ML385. (d) Quantification of the cyto-Nrf2 expression (n = 6). Data are presented as the means ± SEM. ∗P < 0.05 and ∗∗P < 0.01 compared with the control group; #P < 0.05 and ##P < 0.01 compared with the OxyHb group; ††P < 0.01 compared with the OxyHb+Omav group. Supplementary Figure 3: (A) quantification of the number of cells expressing iNOS. (b) Quantification of number of Iba1-positive cells (n = 6). (c) Quantification of the count of cells expressing Arg1. (d) Quantification of count of Iba1+ cells (n = 6). Data are presented as the means ± SEM. ∗P < 0.05 and ∗∗P < 0.01 compared with the Control group; P < 0.05 and P < 0.01 compared with the OxyHb group; ††P < 0.01 compared with the OxyHb+Omav group. Supplementary Figure 4: (A) immunofluorescence staining for Iba-1 (green) and iNOS (red) in the ipsilateral basal ganglia region 1 day after ICH; the nuclei were stained with DAPI (blue); scale bar: 50 μm. (B) Immunofluorescence staining for Iba-1 (green) and Arg1 (red) in the ipsilateral basal ganglia region 1 day after ICH; the nuclei were stained with DAPI (blue); scale bar: 50 μm. (c) Quantification of the ratio of microglia expressing iNOS. (d) Quantification of the ratio of microglia expressing Arg1. (E–F) The concentrations of IL-1β and TNF-α in perihematoma 1 day after ICH were detected using ELISAs (n = 6). Data are presented as the means ± SEM. ∗P < 0.05 and ∗∗P < 0.01 compared with the control group; ##P < 0.01 compared with the OxyHb group; †P < 0.05 and ††P < 0.01 compared with the OxyHb+Omav group. [file 4564471.f1.zip › Supplemental Figure 4.pdf]

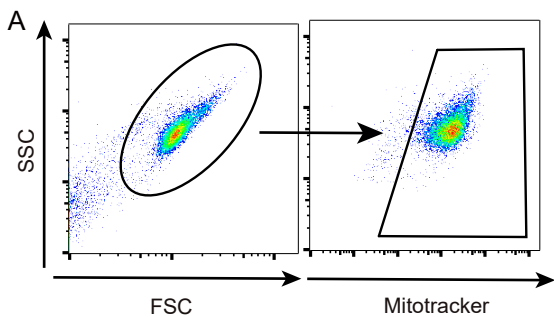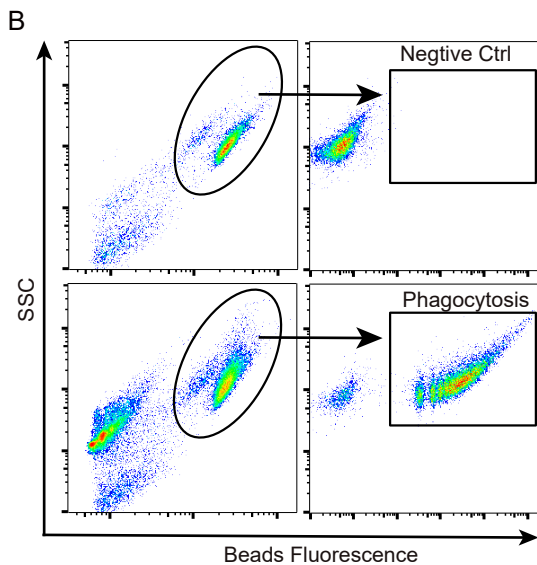

Supplement: Supplementary Materials — Supplementary Figure 1: (a) gating strategy for FCM in mitochondrial bioactivity detection. (b) In vitro phagocytosis gating strategy. Supplementary Figure 2: (a) western blots showing the levels of Nrf2 in BV2 cells treated with vehicle and ML385 under the stress of OxyHb. (b) Quantification of the Nrf2 expression (n = 6). (c) Western blots showing the levels of cytoplasmic Nrf2 in BV2 cells treated with vehicle, OxyHb, Omav, and ML385. (d) Quantification of the cyto-Nrf2 expression (n = 6). Data are presented as the means ± SEM. ∗P < 0.05 and ∗∗P < 0.01 compared with the control group; #P < 0.05 and ##P < 0.01 compared with the OxyHb group; ††P < 0.01 compared with the OxyHb+Omav group. Supplementary Figure 3: (A) quantification of the number of cells expressing iNOS. (b) Quantification of number of Iba1-positive cells (n = 6). (c) Quantification of the count of cells expressing Arg1. (d) Quantification of count of Iba1+ cells (n = 6). Data are presented as the means ± SEM. ∗P < 0.05 and ∗∗P < 0.01 compared with the Control group; P < 0.05 and P < 0.01 compared with the OxyHb group; ††P < 0.01 compared with the OxyHb+Omav group. Supplementary Figure 4: (A) immunofluorescence staining for Iba-1 (green) and iNOS (red) in the ipsilateral basal ganglia region 1 day after ICH; the nuclei were stained with DAPI (blue); scale bar: 50 μm. (B) Immunofluorescence staining for Iba-1 (green) and Arg1 (red) in the ipsilateral basal ganglia region 1 day after ICH; the nuclei were stained with DAPI (blue); scale bar: 50 μm. (c) Quantification of the ratio of microglia expressing iNOS. (d) Quantification of the ratio of microglia expressing Arg1. (E–F) The concentrations of IL-1β and TNF-α in perihematoma 1 day after ICH were detected using ELISAs (n = 6). Data are presented as the means ± SEM. ∗P < 0.05 and ∗∗P < 0.01 compared with the control group; ##P < 0.01 compared with the OxyHb group; †P < 0.05 and ††P < 0.01 compared with the OxyHb+Omav group. [file 4564471.f1.zip › Supplemental Figure1.pdf]
